# Efficacy of a mixture of probiotic agents as complementary therapy for chronic functional constipation in childhood

**DOI:** 10.1186/s13052-017-0334-3

**Published:** 2017-03-07

**Authors:** Marina Russo, Francesca Paola Giugliano, Paolo Quitadamo, Valeria Mancusi, Erasmo Miele, Annamaria Staiano

**Affiliations:** Department of Translational Medical Science, Section of Pediatrics, “Federico II” University of Naples, Via S. Pansini, 5, 80131 Naples, Italy

**Keywords:** Constipation, Probiotics, Polyethylene glycol

## Abstract

**Background:**

About 30% of constipated children continue to struggle with constipation beyond puberty. Growing interest has recently raised on the use of probiotics as complementary therapy for FC, in order to prevent the possible PEG-related intestinal dysbiosis. Our study aimed at evaluating the effect on childhood FC of a probiotic mixture (PM), including Bifidobacteria breve M-16 V®, infantis M-63®, and longum BB536®.

**Methods:**

Fifty-five consecutive children suffering from FC were randomly assigned into two groups: group A received a daily oral combination of PEG plus PM and group B received oral PEG only. Physical and clinical data were collected from each patient at week-1, week-2, week-4, and week-8.

**Results:**

After 1 month, children who experienced improvement in the PEG and in the PEG + PM group were 88 and 81.8%, respectively (*p* = 0.24). After 1 month from the end of the study treatment, a positive trend towards a higher rate of clinical remission was observed within children treated with PM compared to those who took only PEG (percentage of children off therapy: 64 vs 52, respectively; *p* = 0.28).

**Conclusions:**

PEG and PEG + PM are equally effective and safe in the treatment of children with chronic constipation. Nevertheless, further studies are needed to show if adding Bifidobacteria strains to conventional therapy may lead to a better long-term outcome.

## Background

Functional constipation (FC) is one of the most common gastrointestinal (GI) disorders in childhood, with a reported prevalence of 3% in Western countries [1]. FC accounts for about 95% of pediatric chronic constipation, whereas an organic cause, such as structural, endocrine or metabolic disease, can be found in a small minority of patients. The pathophysiology underlying FC is multifactorial and currently not fully understood, even if a withholding behavior following painful defecation is considered one of the main factors leading to the onset of the disorder [2].

The recommended treatment for FC includes a combination of dietary interventions, toilet training and oral laxatives [3]. Although multiple laxatives have been routinely used in the treatment of childhood constipation, recent evidence suggests that polyethylene glycol (PEG) should be the laxative of first choice in pediatrics [4–6]. PEG is a soluble, inert polymer that is not absorbed and acts by osmosis and volume expansion in the large intestine. Long-term treatment with PEG is believed to be safe [7, 8]. Nevertheless, over the last years several studies showed that PEG may change the intestinal milieu by accelerating the passage of luminal contents and by increasing the luminal water content, possibly leading to a change in the intestinal microflora [9]. Changes in microbial community structure related to PEG-induced osmotic diarrhea are profound and show similarities to those observed in other GI disorders including inflammatory bowel diseases [10]. Furthermore, despite the acknowledged short-term efficacy of the available treatments, about 30% of constipated children continue to struggle with constipation beyond puberty [11]. Therefore, growing interest has recently raised on the use of probiotics as complementary therapy for FC. Modulating the GI flora, as a means of improving symptoms and increasing PEG efficacy, may possibly be an attractive treatment option.

The main aim of the present study was to evaluate the efficacy of a probiotic mixture (PM), including Bifidobacteria breve, infantis, and longum added to oral PEG compared to the traditional therapy with PEG alone on childhood FC. Secondary aims were to assess safety and tolerability of the study products for short-term treatment.

## Methods

All consecutive children aged 4–12 years suffering from FC were enrolled from January 2014 to December 2014 at the Gastrointestinal Endoscopy and Motility Unit of the Department of Translational Medical Science, Section of Pediatrics, University of Naples “Federico II”, Italy, until reaching the planned sample size. FC was diagnosed according to the Rome III Criteria as having at least two of these symptoms: < 3 defecations per week; history of excessive stool retention and painful or hard bowel movements; faecal incontinence >2 times/week; withholding behaviour; presence of a large fecal mass in the rectum; history of large-diameter stools [2]. Children with suspected or proved organic causes of constipation, such as Hirschsprung’s disease, spinal bifida, hypothyroidism or other metabolic or renal abnormalities, and mental retardation were excluded from the study. An informed consent was obtained at enrollment from parents of all children younger than 10 years and from both parents and children, if older than 10 years. The study was approved by the Independent Ethics Committee of the “Federico II” University of Naples (reference number: 107/13).

At enrollment, frequency of bowel movements, stool consistency according to the Bristol stool form scale (BSFS) [12], presence of fecal incontinence, abdominal pain, painful defecation, and rectal bleeding were accurately recorded. A thorough medical history was collected by one of the authors and all patients underwent a clinical evaluation, including anorectum digital examination, in order to evaluate whether an abdominal or rectal fecal mass was present. All the enrolled children were then randomly assigned into two groups according to an automatically generated randomization list: group A received a daily oral combination of PEG 4000 (Pergidal® sachets 3.6 g) plus a PM including Bifidobacteria breve M-16 V®, infantis M-63®, and longum BB536® (Tribif® sachets 3 g) (Valeas®Spa, Milan, Italy) and group B received oral PEG only (Pergidal® sachets 3.6 g). The starting dose of PEG was 0.4 g/kg/day for both groups. Increased doses up to 0.8 g/kg body weight daily were allowed by the authors for children not improving after at least 3 days of treatment. The duration of the treatment was 8 weeks. The investigators, the children, and their parents were aware of the study group assignment. Children of both groups underwent rectal disimpaction by rectal enema (120 mL sodium-dioctylsulfosuccinate and sorbitol) on three consecutive days to achieve an empty rectum before starting the treatment trial. The use of other laxatives was not allowed during the study period, whereas enemas were permitted only when there was no defecation for >3 days, as a rescue therapy. A proper toilet training, with regular stool sittings for 5–10 min after each meal, was required. During the 8 weeks of study treatment, the patients and their parents were asked to keep a stool diary, which weekly reported frequency of bowel movements, stool consistency measured through the BSFS, episodes of fecal incontinence, abdominal pain, painful defecation, rectal bleeding, and possible use of enemas.

Follow-up visits were scheduled at week 1 (T1), week 2 (T2), week 4 (T3), week 8 (T4), and week 12 (T5). At each visit, the interim history was assessed, stool diaries were reviewed and discussed, and a further physical evaluation was performed. Clinical progress and compliance with the treatment program were assessed from the stool diaries and history. Week-1 follow-up visit served only to check children’s compliance to the assigned treatment and to allow eventual dose modifications. At week 12 children were re-assessed in order to investigate whether they were still on- or off-therapy. If a child did not return for a planned follow-up visit, follow-up data were obtained through a telephone call by the authors, who gave advice regarding dose adjustment and toilet sitting, and encouraged the parents to come for a follow-up visit if the child had not already recovered.

Primary outcome measures were frequency of bowel movements per week, stool consistency, presence of abdominal pain, faecal incontinence, painful defecation, and rectal bleeding. Treatment success was defined as ≥3 defecation per week, stool consistency ≥ grade 3 on BSFS, and no episodes of abdominal pain, faecal incontinence, painful defecation, and rectal bleeding. Secondary outcome measures were safety and tolerability of the study products evaluated through the incidence of adverse effects such as vomiting, nausea or meteorism, flatulence, and diarrhea.

Data were entered into Excel (Microsoft, Redmond, WA) and analyzed with SPSS software, version 8.0 (SPSS, Chicago, Illinois). Efficacy analyses, bowel movement frequency, stool consistency, and presence/absence of abdominal pain, pain on defecation, fecal bleeding, and fecal incontinence were calculated from the available follow-up data. Our hypothesis was that PEG + PM would have been more successful tham PEG alone in treating chronic FC. Comparisons were made between the initial data and the 2-, 4-, and 8-week follow-up data within each group, and between the two study groups. Statistical analyses included determination of means and SDs, *t* test, *χ*2 test, and Fisher’s exact test, with significance accepted at the 5% level. Results are expressed as mean ± SD or percentage. The power evaluation for both univariate and multivariate tests has been computed with the SPSS Multivariate Anova: population rate, 2.9%; smallest difference, 15%; first type error, 0.05; second type error, 0.05; *p* < 0.05; power, 85%; case/control, 1/1.

## Results

### Initial patient characteristics

**Fig. 1:**
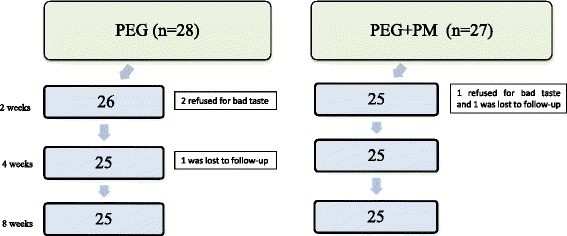
Flow-chart of the study

A total of 62 children and their families were asked to participate in the study. Fifty-five children (26 boys; mean age ± SD: 7.2 ± 2.3 years; age range: 4.1–11.8 years) and their families agreed to participate and were enrolled in the study. According to the randomization list, 28 children (13 boys) were randomly assigned to receive PEG and 27 children (13 boys) to receive an oral combination of PEG plus the probiotic mixture (PEG + PM). Initial patient characteristics of the children who received PEG and PEG + PM are shown in Table 1. The baseline characteristics of the two groups were not statistically different, with respect to demographic features and examined parameters of constipation. During the treatment period, 5/55 (9.1%) children dropped out from the study due to different reasons. A detailed flow diagram of the children’s progress throughout the study with time and reasons for the dropouts is presented in Fig. 1. The final data set of patients completing the study consisted of a total of 50 children (25 in the PEG group and 25 in the PEG + PM group).

**Table 1:** Baseline features of the enrolled children

	PEG (*n* = 28)	PEG + PM (*n* = 27)	*P*
Age, mean ± SD, y	7.1 ± 2.5	7.4 ± 2.8	NS
Male, *n* (%)	13 (46.4)	13 (48.1)	NS
Bowel movements, mean ± SD, episodes per week	2.5 ± 1.1	2.3 ± 0.7	NS
Stool consistency, mean ± SD, BSFS grade	2.6 ± 0.6	2.5 ± 0.7	NS
Presence of fecal incontinence, *n* (%)	4 (14)	5 (18.5)	NS
Presence of abdominal pain, *n* (%)	17 (60.7)	15 (55.6)	NS
Presence of rectal bleeding, *n* (%)	7 (25)	6 (22)	NS

### Two-week follow-up findings

**Fig. 2:**
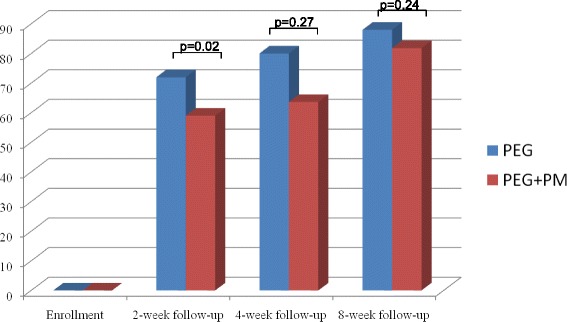
Efficacy of the study treatments (percentages)

In the PEG group 2 children refused to take PEG due to its bad taste. In the PEG + PM group 1 children discontinued participation in the study because of his refusal to take the drug and one child was lost to follow-up. Data of the 2-week follow-up visits are shown in Table 2. An overall improvement of constipation was reported for 72% of children in the PEG group and 59% of children in the PEG + PM group (p:0.02) (Fig. 2). In children of both groups bowel movement frequency increased and stool consistency decreased significantly from baseline (Table 2).

**Table 2:** Frequency of bowel movements and stool consistency at each follow-up

Time points	Bowel movement frequency, mean ± SD, episodes per wk			Stool consistency, mean ± SD, BSFS grade		
	PEG	PEG + PM	*P*	PEG	PEG + PM	*P*
Enrollment	2.5 ± 1.1	2.3 ± 0.7	0.344	2.6 ± 0.6	2.5 ± 0.7	0.395
2-week follow-up visit	5.9 ± 1.3	5.4 ± 1.4	0.168	4.2 ± 0.5	3.9 ± 1.0	0.271
4-week follow-up visit	6.3 ± 0.9	6.0 ± 1.2	0.659	4.4 ± 0.5	4.1 ± 0.6	0.267
8-week follow-up visit	6.3 ± 0.9	6.3 ± 1.0	0.924	4.2 ± 0.5	4.2 ± 0.5	0.857

Both bowel movement frequency and stool consistency were improved significantly at each time point in the PEG and PEG + PM groups, compared with the enrollment values (*p* < 0.05)

### Four-week follow-up findings

One child from the PEG group was lost to follow-up, whereas all children from the PEG + PM group returned for the 4-week follow-up visit. Results of the 4-week follow-up visit are shown in Table 2. The 4-week outcome data were not significantly different between the two treatment groups (p:0.27) (Table 2). In particular, improvement of constipation was reported for 80% of children in the PEG group and 63.6% of children in the PEG + PM group (*p* < 0.05 and *p* < 0.05 respectively, compared with the initial data) (Fig. 2).

### Eight-week follow-up findings

In both the PEG and PEG + PM group all children returned for the follow-up visit. The percentages of children who experienced improvement in the PEG group and the PEG + PM group were 88 and 81.8%, respectively (p:0.24) (Fig. 2). The 8-week data for frequency of bowel movements, stool consistency, fecal incontinence, percentage of children with abdominal pain, rectal bleeding, were not significantly different between the PEG and PEG + PM groups (Table 3). Compared to baseline, both bowel movement frequency and stool consistency improved significantly in children of both groups (Table 2).

**Table 3:** Percentages of abdominal pain, fecal incontinence, and rectal bleeding at each follow-up

	Abdominal pain, %			Fecal incontinence %			Rectal bleeding, %		
	PEG	PEG + PM	*p*	PEG	PEG + PM	*p*	PEG	PEG + PM	*p*
Initial visit	60	56	0.778	14	18.5	0.215	25	22	0.86
2-week visit	16	13	0.534	8	12	0.533	10	8	0.671
4-week visit	12	10	0.778	4	8	0.351	3	3	0.949
8-week visit	8	4	0.369	4	6	0.65	1	2	0.505

### Twelve-week follow-up findings

All children returned for the last assessment or were, alternatively, contacted by telephone. Among the PEG group 13/25 children (52%) were off therapy compared to 16/25 children (64%) within the PEG + PM group (p:0.28).

### Treatment doses

The mean PEG treatment dose in the PEG group was 0.69 g/kg body weight daily at the 2-week follow-up evaluation, 0.73 g daily at the 4-week follow-up evaluation, and 0.71 g/kg daily at the 8-week final evaluation. The mean PEG doses were similar for children who had and had not experienced improvement.

The mean PEG in the PEG + PM group treatment dose was 0.74 g/kg daily at the 2-week follow-up evaluation, 0.77 g/kg body weight daily at the 4-week follow-up evaluation, and 0.75 g/kg at the 8-week final evaluation. The mean PEG + PM doses were similar for children who had and had not experienced improvement. During the study period none of the children needed an enema as rescue therapy.

### Adverse effects

No significant clinical adverse effects were reported with either PEG or PEG + PM except for transient diarrhea, which disappeared with dose reduction. There were no complaints of abdominal distention, increased flatus, or new onset of abdominal pain. The children in both groups who came for follow-up evaluations continued to grow in weight and height, along their growth curves, during the entire study period. There were no new abnormal physical findings on examination.

### Patient acceptance

Several children complained about the taste of PEG and PEG + PM. Nevertheless, only 2/28 (7.1%) and 1/27 (3.7%) children definitely refused to take PEG and PEG + PM, respectively. (p: 1) (Fig. 1).

## Discussion

In this prospective, randomized study we found that the efficacy of PEG + PM and PEG alone in the short-term treatment of children with chronic FC did not differ significantly. At week-2, week-4, and week-8 follow-up evaluations, similar improvement rates were seen in the PEG + PM and PEG groups, with a significant increase in bowel movement frequency, a significant decrease in stool consistency, and a significant resolution of abdominal pain, painful defecation, rectal bleeding, and fecal incontinence, compared to baseline.

In both groups improvement rates increased steadily during the study period, although children treated with PEG + PM experienced benefit slightly more slowly in the first 2 weeks. Nevertheless, no statistically significant difference in any of the measured outcomes within the two groups was reported at the end of the study treatment period nor at the further assessment after 4 weeks from the end of the study treatment. At this point, the number of children who were off-therapy was higher in the PEG + PM group compared to the PEG group. Even if the difference did not reach a statistical significance, we may hypothesize a possible long-term positive effect on constipation of the PM which deserves further attention. However, according to our overall data, in this study we could not definitely demonstrate the superior efficacy of one treatment option over the other for any of the measured outcomes.

Although constipation is a common clinical problem, reports on the efficacy of probiotics for this disorder are still rather contradictory. *Coccorullo* et al. reported a significant improvement of bowel frequency after the administration of Lactobacillus (L.) reuteri DSM 17938 in infants with chronic FC [13]. In addition, *Sadeghzadeh* et al. showed that a mixture of seven probiotic bacteria, including L. casei, L. rhamnosus, Streptococcus thermophiles, B. breve, L. acidophilus, B. infantis, and L. bulgaricus, had a positive role in increasing the frequency and improving the consistency after 1-month treatment [14]. In another study by *Bu* et al. children with constipation were allocated into three groups, receiving L. casei plus L. rhamnosus, magnesium oxide, and placebo. The results of this study showed that the probiotics were as effective as the magnesium oxide, without entailing its possible side-effects [15]. In a study carried out by *Khodadad* et al. children with constipation received paraffin (1.5 ml/kg/day), a mixture of Lactobacilli and Bifidobacteria, or a combination of the two probiotics plus paraffin [16]. According to their findings, defecation frequency increased significantly in children assuming the probiotic mixture. Nevertheless, no beneficial effects were observed on stool form, fecal incontinence, and painful defecation. In contrast with the previous studies, in 2005 a double-blind, placebo-controlled, randomized trial by *Banaszkiewicz* showed that L. rhamnosus was not effective when added to lactulose in the treatment of children with FC [17]. More recently, *Tabbers* et al. showed that in constipated children the fermented dairy product containing B. lactis strain DN-173 010 did increase stool frequency, but this increase was comparable in the control group [18]. The same results were reported by another open trial which showed, in addition, the efficacy of B. breve in improving stool consistency as well [19]. Finally, a recent review by *Vandenplas* et al. concluded that, although some probiotic strains may be helpful in the treatment of childhood constipation, the design of existing trials has been too heterogeneous to allow strong recommendations and that there is a lack of well-designed high-quality randomized controlled trials concerning probiotic treatment of pediatric FC [20].

The authors of the present study are well aware of some methodological drawbacks. In our opinion the main shortcoming is that we did not perform a blinded study because both investigators and patients were aware of the assigned medication. A blinded design would have been hard to carry out because of the need to increase doses differently between the study medications. Furthermore, we lacked to measure the biochemical profiles of children because mandatory blood testing would have affected recruitment and not all children were brought in for the agreed-upon follow-up visits.

Besides efficacy, we’ve also studied the possible adverse effects and patient acceptance to the proposed drugs, which are two further important issues for an appropriate treatment. Both PEG + PM and PEG were not associated with any significant clinical adverse effects and appeared to be safe for oral use in children. Indeed, this finding has already been reported about PEG and PM alone [11, 14]. Compliance with taking the prescribed compounds was similar for children treated with PEG (92.8%) compared with children treated with PEG + PM (96.2%) during the entire 8-week study period. Both medications were administered orally in the form of soluble powder that could be mixed in a beverage of the patient’s choice. Compliance rates are of paramount importance since patient acceptance is a crucial factor for successful long-term resolution of constipation.

## Conclusions

In conclusion, this prospective, randomized, controlled trial showed that adding a mixture of B. breve, B. infantis and B. longum to PEG as complementary therapy for childhood FC confers no additional short-term effect on the main complained symptoms. According to our findings, neither bowel frequency nor stool consistency were significantly altered by the assumption of the PM. Nevertheless, we may hypothesize that adding Bifidobacteria strains to conventional therapy may lead to a better long-term outcome and a possible longer treatment with PM could optimize the efficacy of PEG therapy. In addition, symptoms related to constipation, such as fecal incontinence, abdominal pain, painful defecation, and rectal bleeding, decreased similarly in children assuming PEG with or without PM. As previously mentioned, there are currently conflicting evidence in literature about the use of probiotics for FC. Although experimental models have shown that Bifidobacteria improve colonic peristalsis thus having potential utility for constipation treatment, we have reported that they lack a clinical impact in the short-term therapy of constipated children. Many factors could be involved in their poor efficacy, most of which are yet to be understood. Nevertheless, in our opinion, one of the main issues accounting for our finding concerns the low prevalence of slow transit constipation in pediatric age. Indeed, most constipated children have been shown to have a normal transit time, being rectal obstruction the cause of their disorder. These patients are likely to be less affected by the assumption of probiotics which act by decreasing luminal pH and enhancing colonic transit. Moreover, other factors which could have been involved in the lack of probiotic supplementation efficacy are: the optimal achieving of the primary outcome in the both groups, the lack of fecal microbiota assessment, and the short-term follow-up. Further studies evaluating possible PEG-induced changes in fecal microbiota with longer patient follow-up are welcomed in order to clarify the true role of PEG dysbiosis and possible probiotic treatment.

## Abbreviations

FC: Functional constipation
GI: Gastro-intestinal
PEG: Polyethylene glycol
PM: Probiotic mixture
